# Novel Multifunctional Epoxy (Meth)Acrylate Resins and Coatings Preparation via Cationic and Free-Radical Photopolymerization

**DOI:** 10.3390/polym13111718

**Published:** 2021-05-24

**Authors:** Paulina Bednarczyk, Izabela Irska, Konrad Gziut, Paula Ossowicz-Rupniewska

**Affiliations:** 1 Department of Chemical Organic Technology and Polymeric Materials, West Pomeranian University of Technology, 71-065 Szczecin, Poland; konrad.gziut@zut.edu.pl (K.G.); paula.ossowicz@zut.edu.pl (P.O.-R.); 2 Department of Materials Technology, West Pomeranian University of Technology, 70-310 Szczecin, Poland; izabela.irska@zut.edu.pl

**Keywords:** epoxy (meth)acrylates, coatings, photopolymerization

## Abstract

In this work, a series of novel multifunctional epoxy (meth)acrylate resins based on a low-viscosity aliphatic triepoxide triglycidyl ether of trimethylolethane (TMETGE) and acrylic acid (AA) or methacrylic acid (MMA) have been synthesized. Thanks to the performed modification, the obtained prepolymers have both epoxides as well as carbon–carbon double bonds and differ in their amount. The obtained results indicate that the carboxyl-epoxide addition esterification occurs in the presence of a catalyst (triphenylphosphine) at a temperature of 90 °C, whilst the required degree of conversion can be achieved simply by varying both the reagents ratio and reaction time. The structure of synthesized copolymers was confirmed by spectroscopic analyses (FT-IR, ^1^H NMR, ^13^C NMR) and studied regarding its nonvolatile matter content (NV), acid value (PAVs), as well as its epoxy equivalent value (EE). Due to the presence of both epoxy and double carbon–carbon pendant groups, one can apply two distinct mechanisms: (i) cationic ring-opening polymerization or (ii) free-radical polymerization to crosslink polymer chains. Synthesized epoxy (meth)acrylate prepolymers were further employed to formulate photocurable coating compositions. Hence, when cationic photoinitiators were applied, polyether-type polymer chains with pending acrylate or methacrylate groups were formed. In the case of free-radical polymerization, epoxy (meth)acrylates certainly formed a poly(meth)acrylate backbone with pending epoxy groups. Further, photopolymerization behavior and properties of cured coatings were investigated regarding some structural factors and parameters. Moreover, reaction rate coefficients of photo-cross-linking by both cationic ring-opening and free-radical photopolymerization of the received epoxy (meth)acrylate resins were determined via real-time infrared spectroscopy (RT-IR). Lastly, basic physicomechanical properties, such as tack-free time, hardness, adhesion, gloss, and yellowness index of cured coatings, were evaluated.

## 1. Introduction

Photopolymerization is a method that allows the transformation of a liquid polymerizable composition into a solid in the blink of an eye simply through its exposure to electromagnetic radiation (most often UV). The global UV-curable resins and formulated products market (oligomers, monomers, photoinitiators, and additives) was valued at USD 4.27 billion in 2019 and is projected to reach USD 8.66 billion by 2027 [1]. The interest of industries and researchers in photopolymerization cannot be particularly surprising. Photopolymerization, concerning other analogous methods used in the chemistry of polymers, distinguishes several important advantages. The energy of a mole of photons, at a wavelength of 365 nm, is 130 times greater than the energy that can be provided by a thermal bath at a temperature of 25 °C [2]. Thus, for example, the process of breaking the bond followed by polymerization can be immediately initiated after irradiation, even at room temperature. In addition, the energy of the photons can be accepted by certain light-gathering molecules (chromophores), as opposed to thermal energy, which is transferred to the entire reaction mixture [3]. As a result, in photochemical systems, molecules can be selectively excited and, in turn, reactions are activated “on-demand”. At the same time, the process provides the option of desirable process control by simply turning the light on and off, as well as the ability to spatially control the reaction by masking. Regardless of kinetics, efficiency, and low energy consumption, a common argument in favor of implementing photochemical reactions is the possibility of eliminating solvents from the reaction mixture, as it helps to reduce the release of volatile organic compounds (VOCs) [4]. Due to its advantages, photopolymerization is seen as a pro-ecological technique, especially if cheap light-emitting diodes (LEDs) are used as a source of radiation. Light-induced polymerization is perceived as a modern method of curing because it is most often used to cure thin-film materials such as coatings, adhesives, printing inks [5,6,7,8], and others, such as dental fillings, hydrogels, or 3D-printed items [9,10,11]. Most commonly, depending on the type of photocurable material (resin, monomer, photoinitiator) and thus the emerging active centers that initiate the polyreactions, the reaction proceeds according to a radical or cationic mechanism. Despite various vinyl compounds that polymerize according to the radical mechanism, the majority of UV formulations are based on (meth)acrylates [12]. On the other hand, epoxides, vinyl ethers, and oxetanes are subject to a cationic mechanism [13]. Among others, acrylic and epoxy resins play the most significant role in the production of coating materials [14]. A common problem of radical polymerization is the inhibition of the reaction by molecular oxygen, which, in turn, does not affect the course of cationic polymerization. Therefore, the latter is much better suited to the cross-linking of thicker materials in the air. The termination of cationic polymerization, however, is caused by water vapor, but in a dry environment, termination rates are low. It is worth mentioning that cationic photopolymerization continues in the dark even after the irradiation is stopped [15].

The global market of UV-curable resins is divided into acrylated epoxies, acrylated polyesters, acrylated urethanes, acrylated silicones, and others. The largest share contains epoxy acrylates, which are readily used in UV-curable paints and varnishes [16]. Epoxy acrylic resins are commonly known as vinyl ester resins (VERs) [17]. They are formed by the addition of unsaturated carboxylic acids to the oxirane groups of epoxy resins. Most of the commercially available VERs are made of bisphenol A diglycidyl ether (DGEBA) or bisphenol A diepoxide resins. Due to the addition of acid (usually acrylic or methacrylic), reactive unsaturations are obtained on the VER end-group [18]. The great interest in VERs is caused by the combination of the advantages of epoxy resins, such as excellent mechanical and thermal properties, very good chemical resistance, and good adhesion (caused by the presence of polar hydroxyl and ether groups in the epoxy backbone structure), with the quick hardening of unsaturated polyester resins [19,20].

More and more attention is also paid to so-called hybrid monomers having different types of polymerizable groups in their structure [21]. They also include epoxy acrylates, containing both epoxy and vinyl groups, obtained by modifying a compound with at least two epoxy groups by partially reacting them with (meth)acrylic acid [22,23,24]. Such a structure allows for a choice in the photopolymerization mechanism by selecting a photoinitiator, and thus it is possible to obtain polymers of various types, structures, and properties from the same monomer. By using a radical photoinitiator, a polymer terminated with unreacted epoxy groups can be obtained. If a cationic photoinitiator is used, one may get a structure terminated with unsaturated double bonds. What is more, hybrid compounds also allow for dual curing of materials, usually consisting of radical photopolymerization followed by the thermal hardening of epoxy groups. This curing technology helps to reduce the disadvantages of only using an acrylate resin, e.g., polymerization shrinkage (epoxies exhibit less shrinkage) [25]. Moreover, by dual curing epoxy acrylate resins, one can obtain materials with higher cross-link densities and higher glass transition temperatures [26,27].

The polymers containing reactive pending groups, such as the ones obtained from the hybrid resins, can be applied for the creation of graft copolymers by copolymerization of the pending groups with appropriate monofunctional monomers or can be crosslinked further just by the application of an appropriate complementary initiator [21].

In this study, novel multifunctional epoxy acrylate resins were synthesized, and the effects of the functionality of end-capping groups, such as epoxy or (meth)acrylate groups, on UV-curing behavior and physicochemical properties were investigated. To examine the effects of the amount of epoxy or (meth)acrylate groups, the new epoxy (meth)acrylate resins were synthesized by the addition of acrylic or methacrylic acid to triepoxide triglycidyl ether of trimethylolethane (TMETGE), taking into account the different molar ratio of acid to epoxy groups. Four new trifunctional epoxy (meth)acrylate resins were obtained, which contained at least one epoxy group and at least one acrylate group. The structure of the obtained resins was confirmed by FT-IR and NMR methods. The synthesized resins were investigated as compositions with a cationic or radical photoinitiator. After making the thin polymeric film, UV-curing behavior was investigated using two different methods (Fourier transform infrared (FT-IR), and photo-differential scanning calorimetry (photo-DSC)), then the conversion and the curing rate were compared. The tack-free time, pendulum hardness adhesion, gloss, and yellowness index were measured to evaluate the physicochemical properties of cured coatings.

## 2. Materials and Methods

### 2.1. Materials

The industrial-grade, low-viscosity aliphatic triepoxidetriglycidyl ether of trimethylolethane (Erisys GE-31), abbreviated to TMETGE, was purchased from Huntsman, (Houston, TX, USA). Acrylic acid (AA) and methacrylic acid (MAA), both stabilized with hydroquinone with a purity of 99.5%, was supplied by Acros Organics (Geel, Belgium). Triphenylphosphine (PPh3), Apollo Scientific, Bredbury, UK, was used as a catalyst in the reaction between TMETGE and AA or MAA, while hydroquinone (HQ, Acros Organics, Geel, Belgium) was used as a polymerization inhibitor. All chemicals were employed as received.

The following titration reagents and indicators were used: glacial acetic acid, toluene, potassium hydroxide standard solution 0.1 M in ethanol (KOH) and crystal violet purchased from Chempur (Piekary Śląskie, Poland); chloroform from PPH Stanlab (Lublin, Poland); ethyl alcohol from Avantor (Gliwice, Poland); tetraethylammonium bromide provided by Acros Organics (Geel, Belgium); perchloric acid standard solution 0.1 M in glacial acetic acid supplied by Fischer Chemicals AG (Zurich, Switzerland); phenolophtalein 1% in ethyl alcohol solution from Eurochem BGD (Tarnów, Poland). All chemicals were analytical grade and were used as received.

### 2.2. Synthesis of Multifunctional Epoxy (Meth)Acrylate Resins

**Figure 1:**
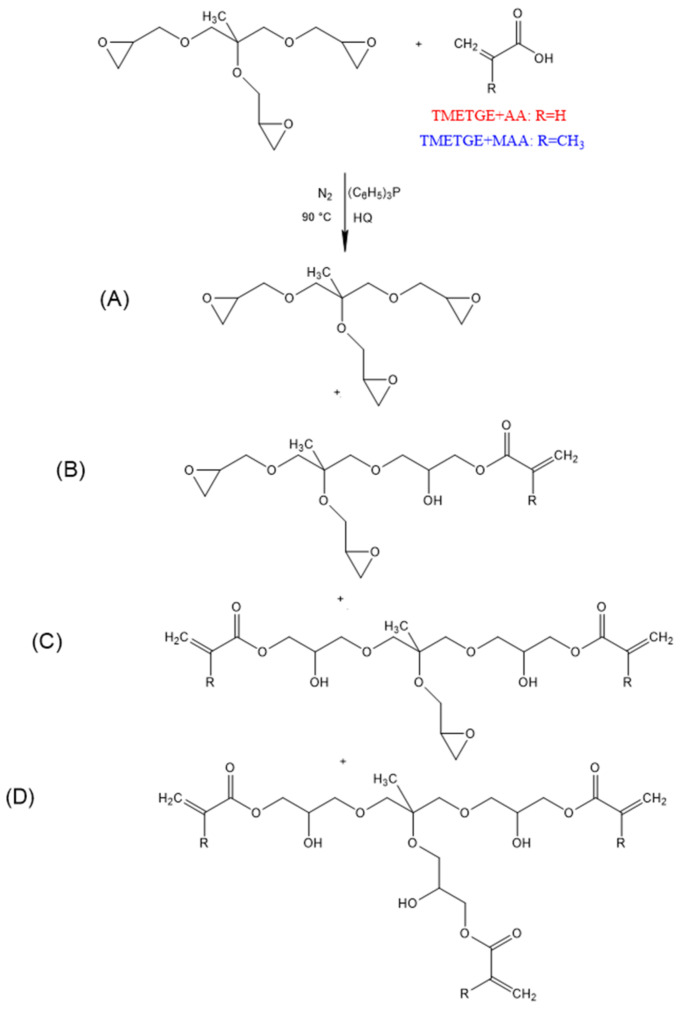
Synthesis of TMETGE-AA and TMETGE-MAA prepolymers by transesterification reactions.

Epoxy acrylates (EAs) were obtained by the addition of acrylic or methacrylic acid to the triglycidyl ether of trimethylolethane. The synthesis conditions were established on the basis of previous works on the synthesis of epox(met)yacrylates by esterification reaction of diglicydyl ether of bisphenol-A [28] or epoxy resin based on bisphenol-A [29,30]. The synthesis was carried out in a 250 mL 3-neck glass reactor (equipped with a thermometer, a condenser, a nitrogen inlet, and a mechanical stirrer), into which TMETGE was introduced. Then, hydroquinone (0.0075 wt.% based on total batch weight) was transferred into the reactor as a radical scavenger at room temperature. Lastly, acrylic/methacrylic acid (0.33 or 0.66 mol relative to resin epoxy value) and catalyst—triphenylphosphine in the amount of 0.9 wt.% (relative to the mass of MAA) were added. All chemicals were used as received, in a single dose, without dissolving. The reaction mixture was heated to 70 °C with vigorous stirring (120 rpm) using an oil bath. Once the homogenous mixture was obtained, the temperature was raised to 90 °C, and the reaction was carried out for 4 to 5 h in a nitrogen atmosphere with stirring. The general reaction scheme, together with the expected products, is presented in Figure 1.

### 2.3. Characterization Methods

The infrared spectra were acquired with a Thermo Nicolet 380 FT-IR spectrometer (Thermo Scientific, Waltham, MA, USA). Sixteen scans were averaged for each sample in the range of 4000–400 cm^−1^, at room temperature.

The prepared compounds were identified by ^1^H NMR and ^13^C NMR. ^1^H-NMR (400.13 MHz) and ^13^C-NMR (100.62 MHz) spectra were recorded in CDCl_3_ on a Bruker Avance III HD 400 spectrometer (Bruker, Billerica, MA, USA). TMS was used as an internal standard.

The nonvolatile matter content (NV) was evaluated thermogravimetrically using a Moisture Analyzer MAX 60/NP (Radwag, Poland), according to ISO 3251:2019 standard. The analysis was performed at 140 °C for 30 min. The sample weight was approximately 1 g. NV (%) = (*m*_2_/*m*_1_) × 100%, where *m*_1_ is the weight of the EA sample; *m*_2_ is the residual weight of the sample after heating.

Partial acid values (PAVs) were determined by colorimetric titration according to EN ISO 2114:2000 standard. The EA samples were dissolved in a mixture of solvents (toluene:ethyl alcohol, volume ratio 2:1), and then titrated with a standard KOH solution (0.1 N) in the presence of phenolphthalein. As the endpoint of the titration, the color change of the solution into pink (lasting from 20 to 30 s) was assumed. PAVs (mgKOH/g) were calculated using the following formula:

(1) $$\mathrm{PAV}=\frac{56.1\left(V_{1}-V_{2}\right)c}{m_{1}}$$

where 56.1 is a constant value (molar mass of KOH, g/mol), *m*_1_ is mass of the analytical sample (g), *V*_1_ is the volume of the KOH solution used to neutralize the resin solution (mL), *V*_2_ is the volume of the KOH solution used for the blank test (mL), and c is a concentration of KOH solution (mol/L).

The partial acid value of solid resin was calculated using the following equation:

(2) $${\mathrm{PAV}}_{s}=\mathrm{PAV}\times \frac{100}{NV}$$

where PAVs is the partial acid number (mgKOH/g) and NV is nonvolatile content (%).

The PAVs values were used to estimate methacrylic acid conversion (MAAC) according to the following equation:

(3) $$MAAC=100-\frac{PAV_{s}\times 100}{PAV_{s0}}$$

where PAV_s0_ is the initial value of PAV_s_ (mg KOH/g).

Epoxy equivalent (EE) was determined by means of colorimetric titration, according to EN ISO 3001:1999 standard. EAs samples were dissolved in chloroform and then glacial acetic acid and tetraethylammonium bromide solution were added. The titration was carried out with the standard solution of chloric(VII) acid (0.1 N) to achieve a stable green color in the presence of crystal violet. EE (g/mol), was calculated according to the equation:

(4) $$EE=\frac{1000m}{\left(V_{1}-V_{0}\right)\left(1-\frac{1-t_{s}}{1000}\right)c}$$

where *m*—the mass of the EA sample (g), *V*_0_—the volume of the perchloric acid solution used in the blank determination (mL), *V*_1_—the volume of the perchloric acid solution used during titration (mL), *t*—the temperature of the perchloric acid solution during the EE determination and the blank test (°C), *t_s_*—the temperature of the chloric acid solution during titer adjustment (°C), and *c*—concentration of a perchloric acid solution (mol/L).

Epoxy group conversion (EGC) was estimated from EE measurements via the following formula:

(5) $$EGC=100-\frac{EE_{0}\times 100}{EE_{measured}}$$

where EE_0_ is the initial value of EE (g/mol) and EE_measured_—EE value at a specific time.

The viscosity tests were carried out using a cone-plate viscometer LAMY RM-100 plus CP 2000.

### 2.4. Preparation of Coating Compositions and Cured Films

The coating compositions have been formulated using epoxy resin or synthesized epoxy acrylates and 3 wt.% PI (bis(dodecylphenyl)iodoniumhexaflouroantimonate in propylene carbonat, Deuteron UV 1240, Deuteron as a cationic photoinitiator or ethyl(2,4,6-trimethylbenzoyl)-phenyl phosphinate, Omnirad TPOL, IGM Resins, as a radical photoinitiator. The maximum absorption characteristics of the cationic photoinitiator (Deuteron 1240) are between 220–250 nm, and the radical photoinitiator is at the wavelength 274, 290, and 370 nm, respectively. The components were stirred together under dark conditions until a homogeneous mixture was obtained. Subsequently, the curing solution was applied to the glass substrates by means of a gap applicator (120 µm). The polymeric film was cured under a light source (UV lamp, Aktiprint-mini 18-2, type: UN50029, Technigraf GmbH, Grävenwiesbach, Germany) at room temperature and irradiated under UV light with an intensity of 200 mW/cm^2^ to dryness.

### 2.5. Characteristics of the Photopolymerization Process and Properties of Cured Coatings

The UV-curing process of epoxy (meth)acrylates was isothermally monitored (25 °C) in a nitrogen atmosphere for 15 min by means of a photo-DSC apparatus (Q100, TA Instruments, New Castle, DE, USA) equipped with the Omnicure S2000 UV light emitter (280–480 nm, 500 mW/cm^2^; Excelitas Technologies, Waltham, MA, USA), providing a first hint about the photoreactivity of the obtained systems. A polymerization solution was composed of epoxy (meth)acrylate resin and 3 wt.% of cationic or radical photoinitiator.

Fourier transform infrared spectra (FT-IR) were obtained on a Nicolet iS5 instrument (Thermo Fisher, Waltham, MA, USA). The resolution is 4 cm^−1^ and the scanning range is 400–4000 cm^−1^. The recording interval of the spectrum was 10 s. Series real-time IR (RT-IR) was used to determine the conversion of epoxide groups of acrylic double bonds. More importantly, this spectroscopic technique permits in situ monitoring of the chemical processes by mimicking the disappearance of the characteristic bonds of the reactive monomer subjected to UV exposure [31]. The mixture of epoxy or epoxy acrylate resins and an initiator was placed in a mold made from glass slides and spacers of 15 mm in diameter and 0.2 mm in thickness. The samples were placed in the compartment of a Fourier transform infrared spectrometer and were simultaneously exposed to a UV light source (mercury UV lamp, 36 W, 280–400 nm, 10 mW/cm^2^) and an IR analyzing light beam. The absorbance change of the epoxide group (C–O) and acrylate double bond (C=C) peak area was correlated to the extent of polymerization. The degree of conversion (DC) can be expressed by the following relations: DC (%) = (A_0_ − A_t_)·100/A_0_, where A_0_ is the initial peak area before irradiation and A_t_ is the peak area at time t. The photopolymerization rate (R_p_) was calculated by the following relations: R_p_ = dDC/d_t_, where t is the time of irradiation [32].

The following tests were performed in order to evaluate the mechanical properties of the cured coatings: tack-free time, pendulum hardness test, adhesion, gloss, and yellowness index. Tack-free time was measured as a surface cure time according to ISO 9117. This is the time at which the coating is deemed to have properly adhered and has achieved the final technical parameters. The hardness of coatings was tested using Persoz pendulum hardness on the glass substrate (TQC Sheen, Capelle aan den IJssel, The Netherlands) according to ISO 1522 standard. Adhesion to glass substrate was evaluated according to PN-EN ISO 2409 (cross-cut method; BYK, Germany). Gloss was measured by spectrometer GLS (SADT Development Technology Co. Ltd., Beijing, China) according to ASTM D523. Yellowness Index is a number calculated from spectrophotometric data that describes the change in color of test samples. This parameter was measured according to ASTM E313 using precision colorimeter NH-145 (3NH Technology Co. Ltd., Shenzhen, China).

## 3. Results and Discussion

### 3.1. The Approach to the Development of the Prepolymers Synthesis

**Figure 2:**
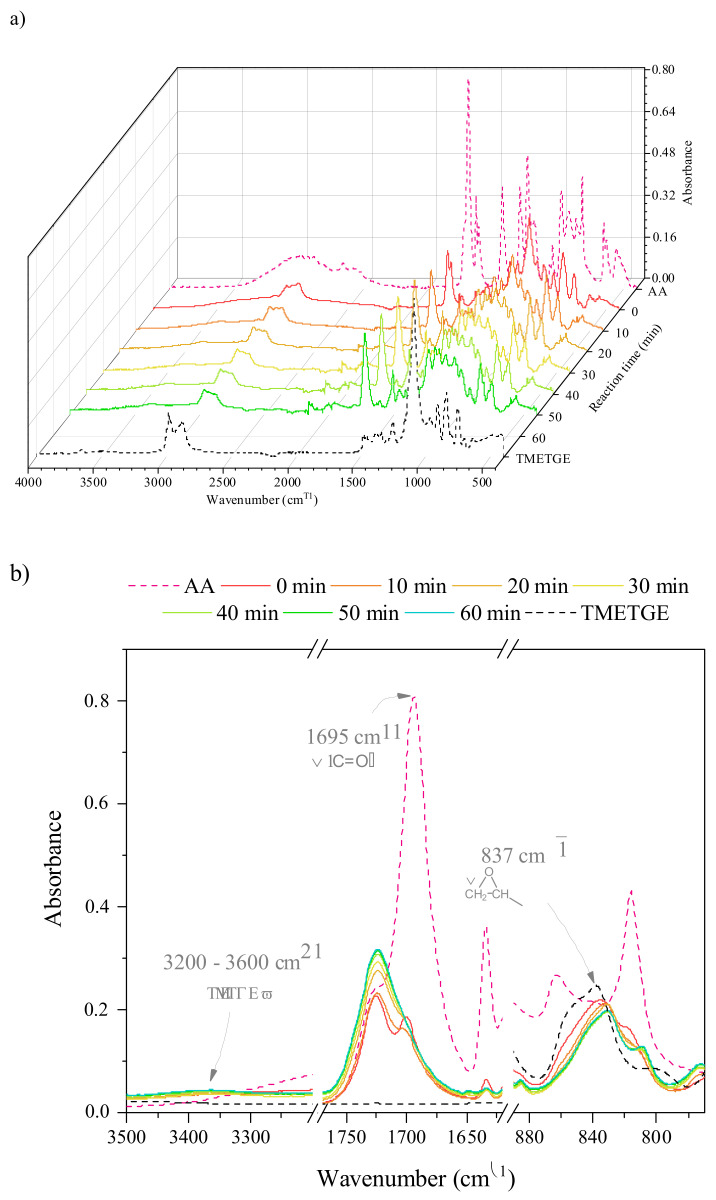
FTIR spectra of TMETGE resin, methacrylic acid, and prepolymers as a function of the reaction time: (**a**) 3D representation, (**b**) an enlarged view of the IR spectra in the range typical for carbonyl, epoxy, and a hydroxyl group.

In order to gain some insight into the reaction course, we performed an FT-IR spectroscopy kinetics study on the model epoxy-acrylate system. A mixture of TMETGE, AA, catalyst, and reaction inhibitor were heated to 90 °C and FT-IR spectra were collected every 10 min for a total of 60 min (stacked together with the reaction substrates at Figure 2a). Regions characteristic to the absorption bands corresponding to the vibrations of carbonyl, epoxy, as well as hydroxyl groups were found to be particularly interesting to observe the progress of the transesterification reaction course (Figure 2b). Absorption bands specific for and C-O-C stretching vibrations of epoxy groups in aliphatic-type di- and triglicydyl ether occur at ~837 cm^−1^ [33], carbonyl stretching mode in AA appears at a wavelength of 1695 cm^−1^ [34,35], whilst additional absorption peak corresponding to stretching vibrations of –O–H groups’ resulting from the epoxide ring-opening develops at 3600–3200 cm^−1^ [33,36,37]. Close inspection of TMETGE-AA prepolymers’ spectra in the ranges described above unambiguously confirms that transesterification reactions took place under applied conditions. In particular, as the reaction time proceeds, an additional absorption peak corresponding to C=O stretching vibrations develops at a wavelength of 1725 cm^−1^, thereby suggesting that ester bonds are formed due to the reaction of carboxyl groups of AA and epoxide groups of TMETGE. This is in good agreement with previous studies reporting the shift of the C=O band position to a higher value of wavenumber after the esterification reaction [38]. Moreover, the intensity of the signal at ~837 cm^−1^ progressively decreases, whilst the intensity of broadband at 3600–3200 cm^−1^ slightly increases, suggesting the consumption of epoxy group (due to epoxide ring-opening) and evolution of additional –O–H groups’, respectively.

### 3.2. Prepolymer Characterization

**Figure 3:**
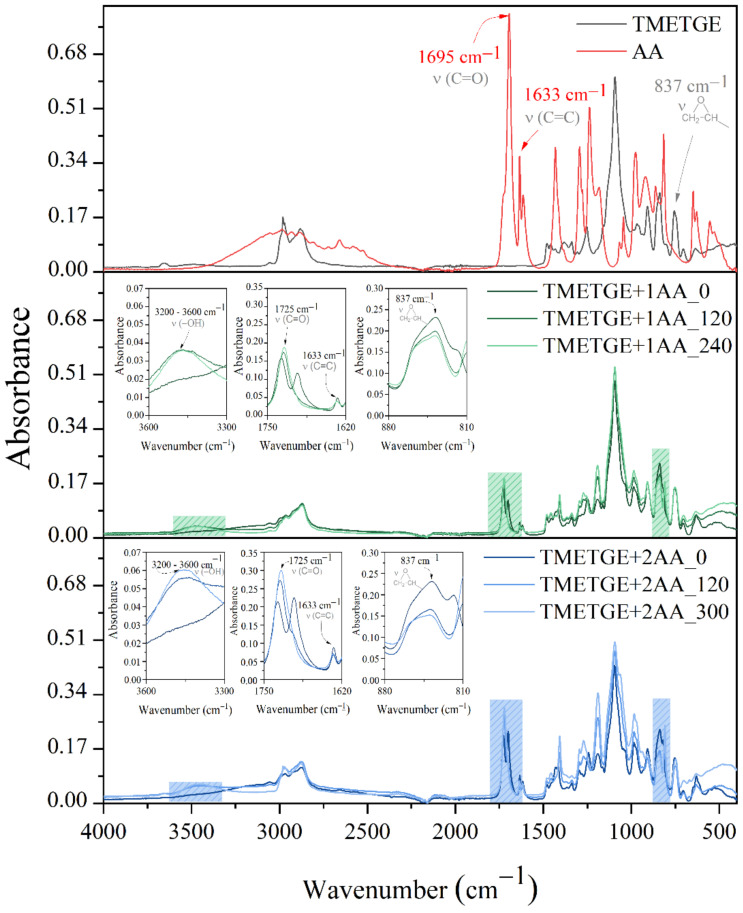
FT-IR spectra of reaction substrates TMETGE and AA (**top panel**), TMETGE + 1AA prepolymers collected throughout the reaction (middle panel) TMETGE + 2AA prepolymers collected throughout the reaction (**bottom panel**). The digit indicates the reaction time; “0” refers to the reaction mixture preheated to 70 °C.

**Figure 4:**
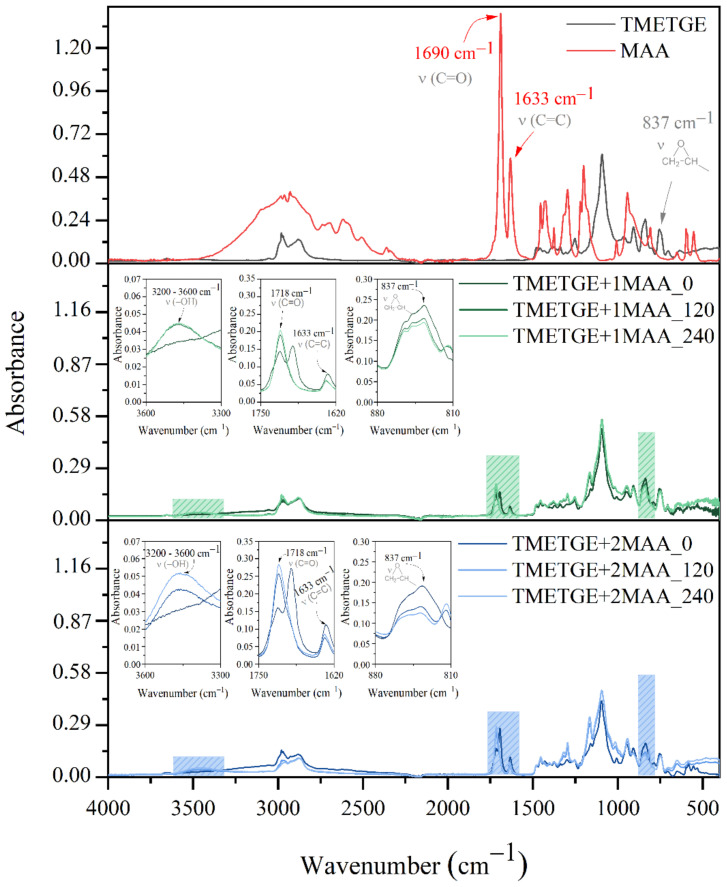
FT-IR spectra of reaction substrates TMETGE and MAA (**top panel**), TMETGE + 1MAA prepolymers collected throughout the reaction (**middle panel**), TMETGE + 2MAA prepolymers collected throughout the reaction (**bottom panel**). The digit refers to the reaction time; “0” stands for reaction mixture preheated to 70 °C.

Even though preliminary RT-IR studies unquestionably confirmed that esterification reactions occur in the TMETGE-AA system, one has to determine the optimum reaction time in all investigated systems using the quantitative method. To study the product evolution over the reaction time simultaneous spectroscopic (FT-IR), titration (PAVs and EE) along with nonvolatile matter content (NV) investigations were performed. The spectra of reaction substrates, TMETGE-AA and TMETGE-MAA prepolymers, are shown in Figure 3 and Figure 4. Table 1 summarizes the obtained values of PAVs, EE, as well as the epoxy group conversion (EGC) and acrylic/methacrylic acid conversion (AAC/MAAC) expressed as the percentage of reacted epoxide groups and the percentage of reacted AA/MAA at a specific reaction time, respectively. The FT-IR spectra of TMETGE-AA prepolymers are consistent with previous observations on the model system (see Section 3.1), revealing the presence of characteristic band absorptions: (i) ~837 cm^−1^, C–O–C stretching vibrations of epoxy groups, (ii) ~1725 cm^−1^, C=O stretching vibrations and (iii) ~3600–3200 cm^−1^, stretching vibrations of -O-H groups’ (see enlarged regions at the middle and bottom panel of Figure 3). The TMETGE-MAA prepolymers spectra are analogous to the ones registered for TMETGE-AA systems except that the absorption bands related to C=O stretching vibrations are seen at 1718 cm^−1^ (Figure 4). The variation in the absorbance intensity of bands mentioned above is clear evidence that transesterification reaction proceeded in both TMETGE-AA and TMETGE-MAA systems until an equimolar degree of conversion has been attained. For example, in the TMETGE-1AA system, the absorbance intensity of the band in the range typical for epoxy groups decreases from 0.25 to 0.20 within 240 min, whilst the same band in TMETGE-2AA has an intensity of 0.14 at the end of the process. On the other hand, the absorbance intensity of the bands of the –O–H and C=O stretching vibrations are exhibiting significantly higher intensity in the TMETGE-2AA system, where the molar ratio of epoxy groups to acid equals 3:2. It is, therefore, reasonable to consider that in the latter system, more epoxy groups undergo ring-opening reactions forming at the same time as the increased amount of new ester bonds and additional hydroxyl groups. These results agree well with titration experiments. Generally, as the reaction time proceeds, an increase in both NV and EE, as well as a decrease in PAVs, can be seen. Moreover, the viscosity of the product increases slightly as a result of reactions occurring in the system. It was found that a product with a viscosity of ~1000 mPa*s can be obtained at epoxy groups/acid molar ratio of 3:1, whilst a molar ratio of 3:2 gives the final product viscosity of ~4000 mPa·s. All the above considerations are also relevant when it comes to the systems based on methacrylic acid (TMETGE-1MAA and TMETGE-2MAA). In all cases, the reaction was conducted until the nearly equimolar AA/MAA was reacted (PAVs < 20 mgKOH/g). At this point, it is worth emphasizing that one has to prolong the reaction time from 240 to 300 min in order to attain sufficient AAC, as the content of AA increases from 1 to 2 moles. Interestingly, the modification of TMETGE with methacrylic acid appeared to be faster, irrespective of the resin to acid molar ratio, an appropriate degree of conversion was achieved within 240 min of reaction. As expected, EE of the final product appeared to be correlated to prepolymer composition, in particular, ~30% and ~60% increase in EE value was recorded after acrylation in the TMETGE-1AA and TMETGE-2AA, respectively. This correlates well with the expected composition and supports the high efficiency of reactions occurring in the system.

**Figure 5:**
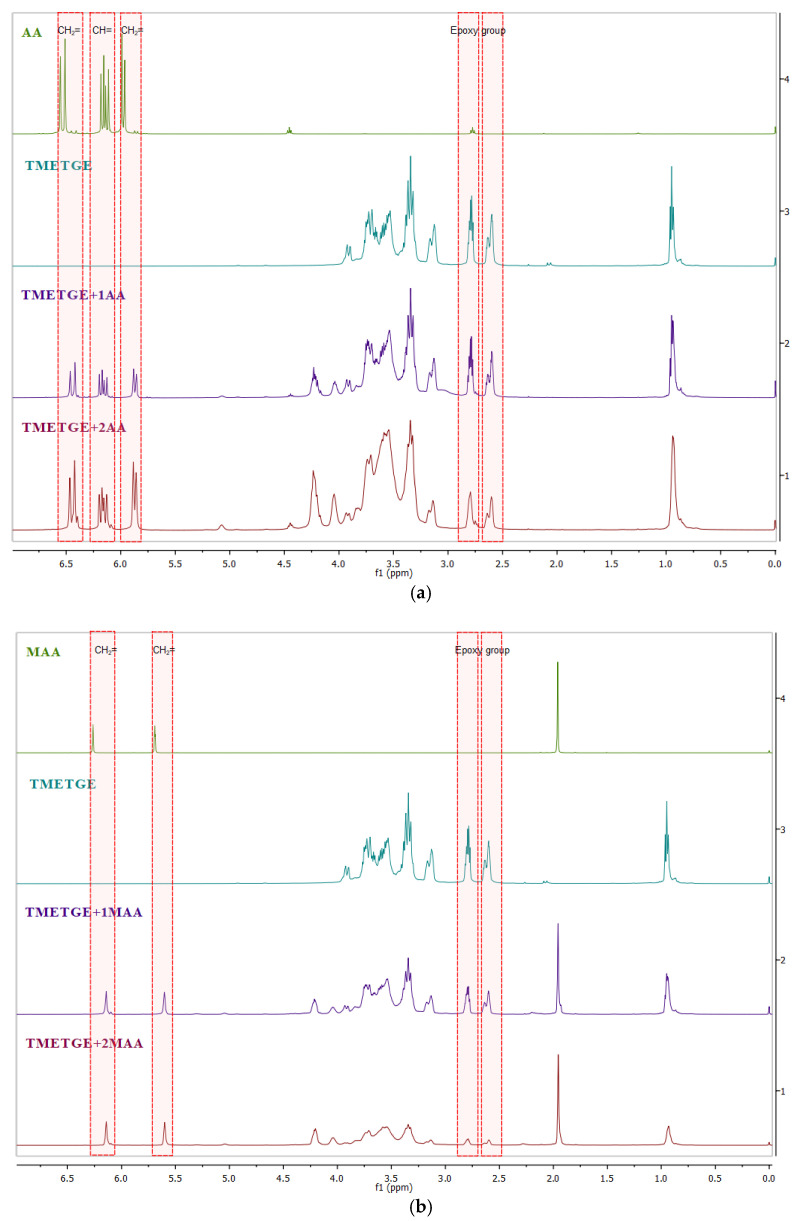
^1^H NMR spectra (CDCl_3_) of (**a**) AA, triglycidyl ether of trimethylolethane, TMETGE + 1AA, and TMETGE + 2AA (from the top) and (**b**) MAA, triglycidyl ether of trimethylolethane, TMETGE + 1MAA, and TMETGE + 2MAA (from the top).

**Figure 6:**
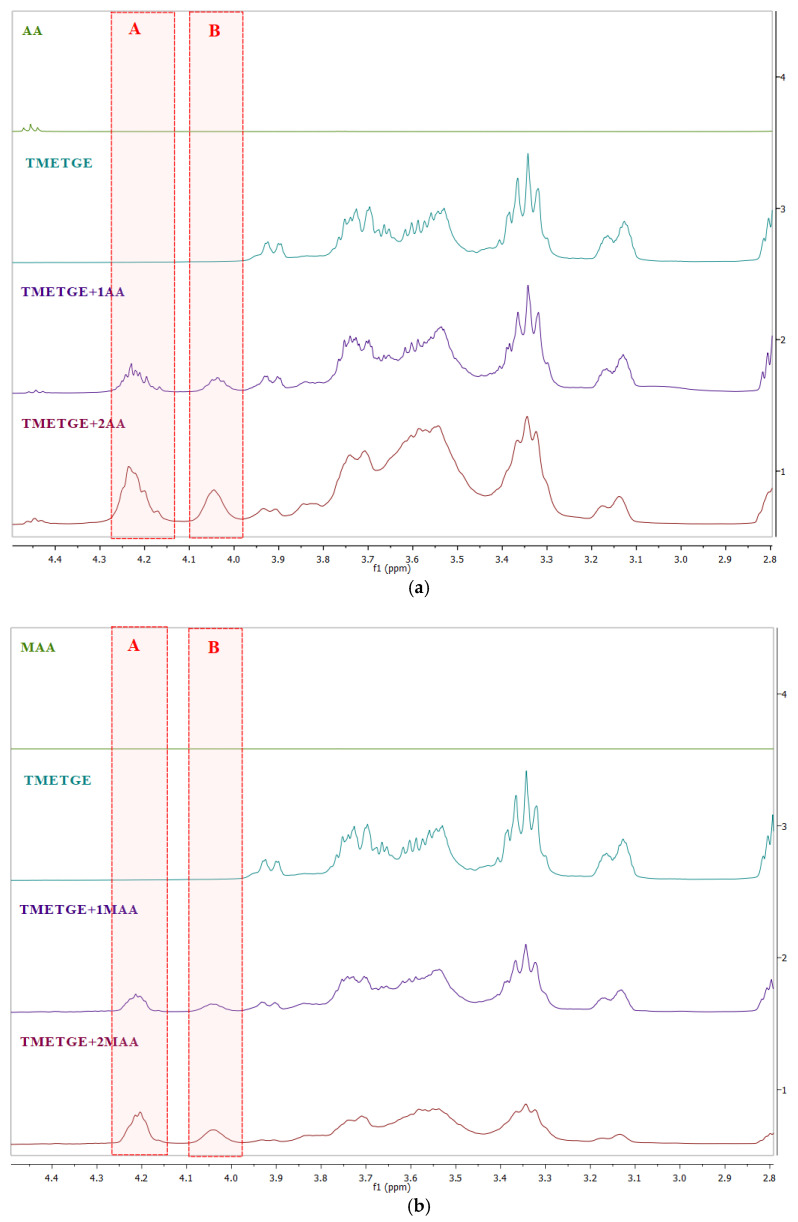
Fragments of the ^1^H NMR spectra (CDCl_3_) of (**a**) AA, triglycidyl ether of trimethylolethane, TMETGE + 1AA, and TMETGE + 2AA (from the top), (**b**) MAA, triglycidyl ether of trimethylolethane, TMETGE + 1MAA, and TMETGE + 2MAA (from the top) showing the formation of new signals.

**Figure 7:**
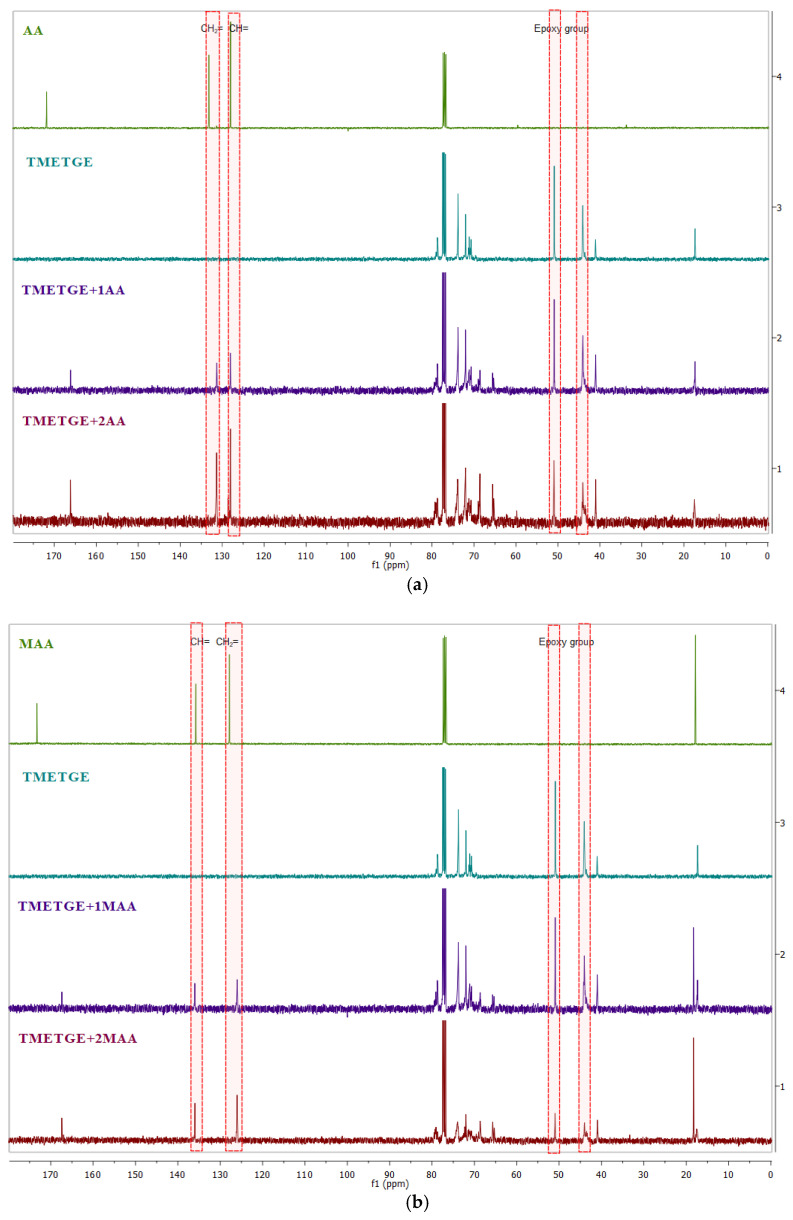
^13^C NMR spectra (CDCl_3_) of (**a)** AA, triglycidyl ether of trimethylolethane, TMETGE + 1AA, and TMETGE + 2AA (from the top) and (**b**) MAA, triglycidyl ether of trimethylolethane, TMETGE + 1MAA, and TMETGE + 2MAA (from the top).

**Figure 8:**
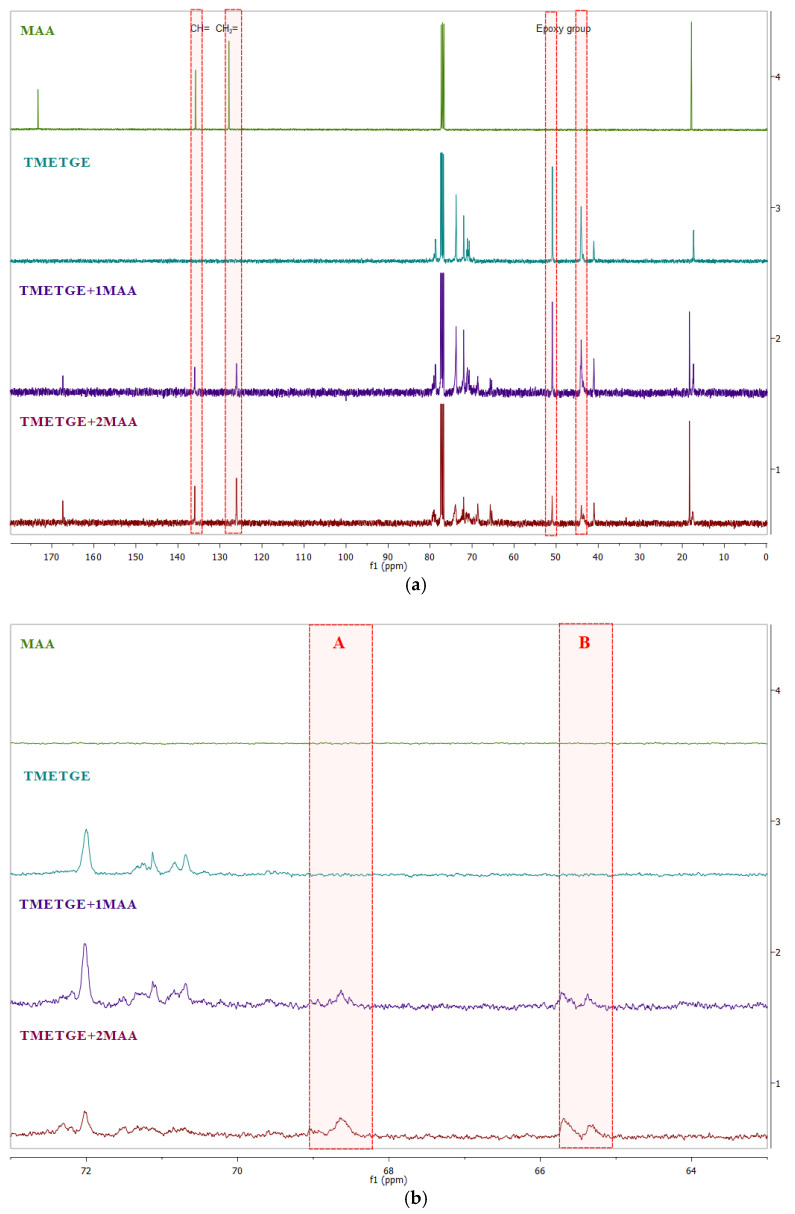
Fragments of the ^13^C NMR spectra (CDCl_3_) of (a) AA, triglycidyl ether of trimethylolethane, TMETGE + 1AA, and TMETGE + 2AA (from the top) and (**b)** MAA, triglycidyl ether of trimethylolethane, TMETGE + 1MAA, and TMETGE + 2MAA (from the top).

**Figure 9:**
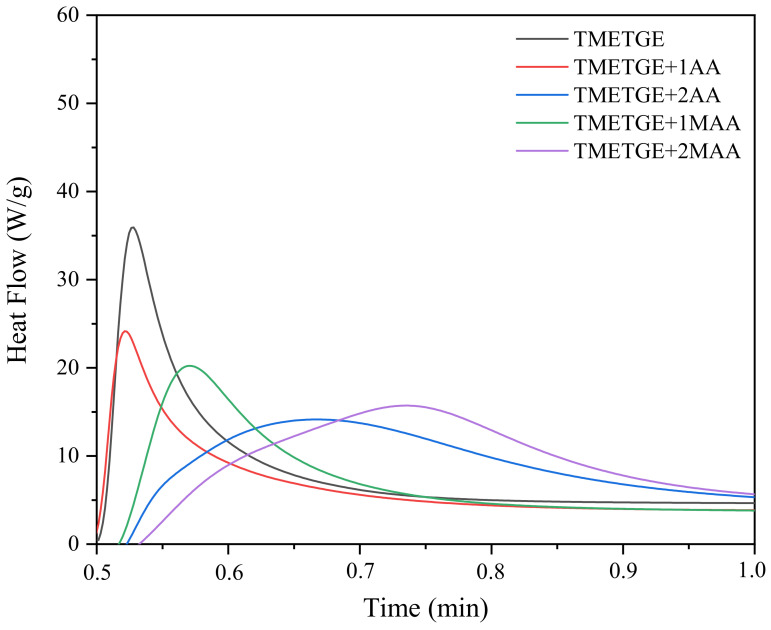
Photo-DSC exotherms for the photopolymerization of epoxy (meth)acrylate formulations with cationic photoinitiator.

In order to confirm the triepoxidetriglycidyl ether of trimethylolethane etherification modification with AA and MAA, the obtained epoxy (meth)acrylic resins were characterized by ^1^H and ^13^C NMR spectra, the results of which are illustrated in Figure 5, Figure 6, Figure 7, Figure 8 and Figure 9. Figure 5 presents a statement of the ^1^H NMR spectra of substrates (acrylic acid or methacrylic acid and triglycidyl ether of trimethylolethane) and obtained products (using an equimolar amount and with twice the molar excess of acrylic acid or methacrylic acid). In Figure 5, signals from protons occurring in unsaturated bonds and epoxy group are marked in red (CH = occurring in the range of 6.15–6.16 ppm, CH_2_ = occurring in the range of 5.87–5.98 ppm and 6.45–6.53 ppm) in acrylates, (CH_2_ = occurring in the range of 5.60–5.69 ppm and 6.14–6.26 ppm) in methacrylates and the epoxy group 2.61–2.62 ppm (–CH_2_-) and 2.79–2.80 ppm (–CH–), respectively. Figure 6 shows a fragment of the ^1^H NMR spectrum, which shows the appearance of new signals as a result of the reaction of the ring-opening of the epoxide (signals A and B). These signals are assigned to the protons of the –CH(OH) (~4.2 ppm) and CH_2_O group (~4.0 and ~4.2 ppm), respectively. At the same time, signals from the vinyl group and epoxy groups are visible.

Obtaining the desired structures was confirmed also by recording ^13^C NMR spectra, which showed the appearance of additional signals (C and D) from the CH_2_O group carbons (~65.5 ppm) and –CH(OH) (~70.0 ppm). The ^13^C NMR spectra of both the substrates and the obtained products are shown in Figure 7, while the fragment of the spectrum where new signals have been marked from the ring-opening reactions are shown in Figure 8. As shown in Figure 7, the spectra of the products contain signals from both the epoxy group and the unsaturated bond. These characteristic signals are marked in red, CH = occurring in the range of 128.00–128.04 ppm, CH_2_ = occurring in the range of 133.17–131.36 in acrylates, 126.01–127.86 ppm and 135.74–135.98 ppm, respectively, in methacrylates, and 44.04–44.08 ppm (–CH_2_–) and 50.87–50.94 ppm (–CH–), respectively, in the epoxy group.

### 3.3. The Photocuring of the Epoxy (Meth)Acrylate Prepolymers

The kinetics of photopolymerization is useful in understanding the rate and degree of curing. It is well known that acrylates and methacrylates do not polymerize by a cationic polymerization mechanism, while epoxy monomers do not polymerize by free-radical means. Hence, when cationic photoinitiators were applied, polyether-type polymer chains with pending (meth)acrylate groups were formed. In the case of free-radical polymerization, epoxy (meth)acrylates certainly formed a polymethacrylate backbone with pending epoxy groups [21].

**Figure 10:**
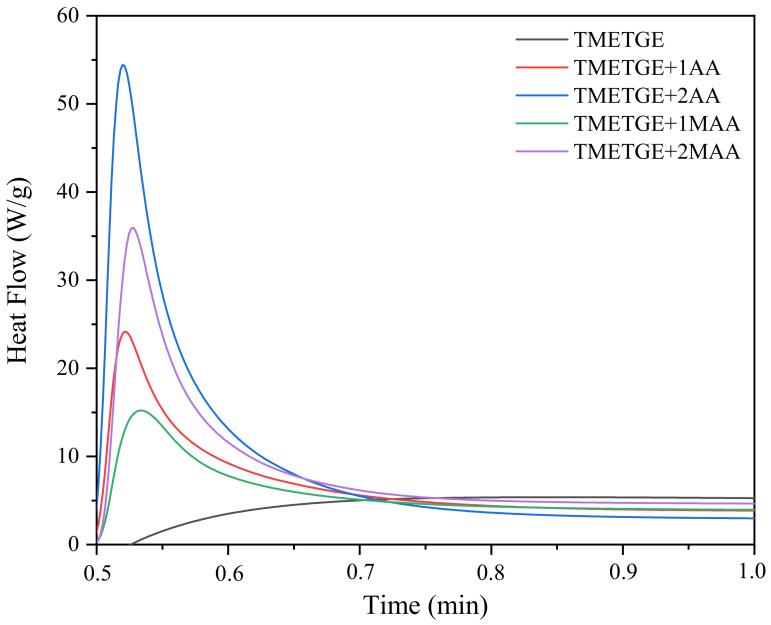
Photo-DSC exotherms for the photopolymerization of epoxy (meth)acrylate formulations with radical photoinitiator.

Figure 9 and Figure 10 shows the photo-DSC exotherms for the photopolymerization of the UV-curable epoxy (meth)acrylate systems containing different photoinitiator. It is obvious from Figure 9 that the system TMETGE containing three epoxide groups in the molecule exhibit higher exotherms as well as much faster polymerization reactivity than for the other systems with two or one epoxide group, which polymerize much slower. Conversely, in the case of formulations containing radical photoinitiator, systems containing two acrylate or methacrylate groups in the molecule polymerize faster (Figure 10). Interestingly, these results indicate that the acrylates polymerize faster than methacrylates.

**Figure 11:**
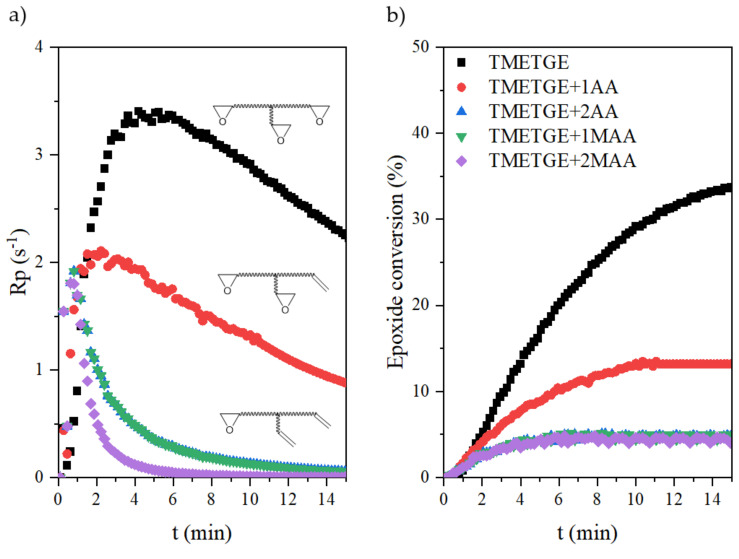
The photopolymerization rate (**a**) and the epoxide conversion (**b**) curves of the epoxy (meth)acrylates.

**Figure 12:**
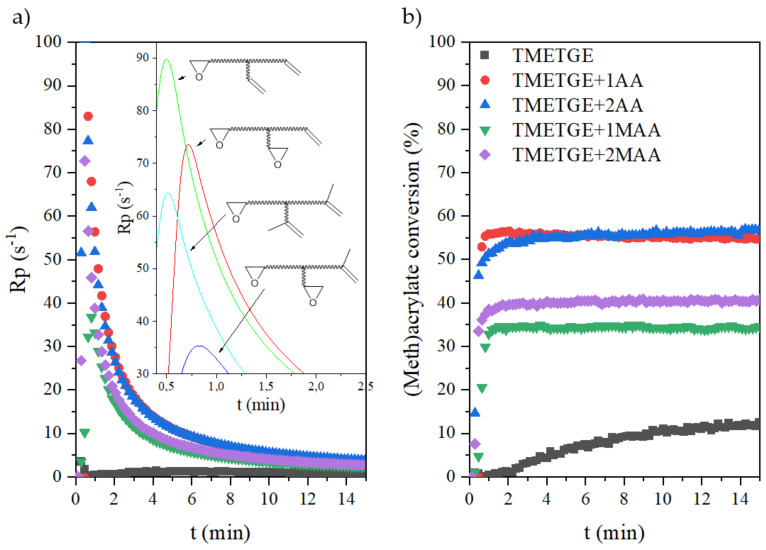
The photopolymerization rate (**a**) and the unsaturated double bond conversion (**b**) curves of the epoxy (meth)acrylates.

Through the RT-IR method, the curves of the photopolymerization rate (R_p_) and conversion of epoxy (meth)acrylates containing different amounts of epoxy, acrylate, or methacrylate groups curing via the cationic or radical process were investigated, as shown in Figure 11 and Figure 12. Photoinitiated cationic ring-opening polymerization of EAs, containing one, two, or three epoxy-terminated groups in the molecule were conducted at room temperature and with access to atmospheric oxygen. The same conditions were also used in photoinitiated radical double bond polymerization of EAs, containing one or two acrylate or methacrylate-terminated groups. As expected, the progressive disappearance of the various IR bands characteristic of the epoxy or acrylate double bonds was observed. The extent of the epoxy groups’ reactions was determined by the areas of the peak at 915 cm^−1^, which is due to C–O stretching in the epoxy ring. Simultaneously, the extent of double bond reactions was determined by the peak areas of the double bond peak at 1635 cm^−1^.

The cationic photopolymerization of neat TMETGE proceeded with the highest rate among the investigated systems in this series, up to 35% conversion, and then underwent a transition into a sluggish propagation with a correspondingly low polymerization rate. The initial high reactivity of TMETGE may be attributed to the high epoxy group content and low viscosity of the system. As the number of epoxy groups decreases and the viscosity of the system increases, the photopolymerization rate is significantly reduced. In addition, the progressive cationic reaction reduces the mobility of the polymer chains, increases the microviscosity of the system, and reduces the speed. Therefore, modification of TMETGE by adding (meth)acryl groups in place of epoxy groups resulted in a reduction of the photopolymerization rate and lower conversion. This is related not only to the reduction in the number of epoxy groups but also to the increase in the viscosity of the system, forming polyether-type polymer chains with pending (meth)acrylate groups. It can be seen that the photopolymerization rate of all of the processes by the cationic mechanism increases to 5 min, while the rate of radical processes experiences a steep increase in the first 2 min and then slows down. Thus, the rate of radical polymerization and formed a polymethacrylate backbone with pending epoxy groups is much higher than the cationic one, which could be attributed to the increase in the decomposition rate of the radical photoinitiator, leading to a larger number of primary radicals in the system at the beginning of the reaction. The higher initiation rate of radical photopolymerization may also be related to the absorption characteristics of the radical photoinitiator used, which completely match the emission characteristics of the UV lamp, especially for the highest intensity bands. In addition, the significant increase in unsaturated double bond conversion might be caused by a temporary excess of free volume due to the high amount of primary radicals. It is well known that, during the reaction of the UV-curing system, the mobility of the polymer chain and the diffusion capacity of the active radicals have a great influence on the conversion efficiency [32]. Epoxy (meth)acrylates with methacrylate groups had a higher viscosity. In high-viscosity systems, the diffusion of primary living free radicals was limited, leading to a reduction in the rate of photopolymerization. In addition, the restricted movement of the double bond due to the presence of an additional methyl group and related spatial hindrance also reduced the complete curing of coatings. Consequently, acrylates have a higher photopolymerization rate compared to methacrylates, and the conversion of unsaturated double bonds increased with the increasing content of active unsaturated double bonds. The reason for this phenomenon is that the system has a higher polymerization rate in the early stage of the reaction. The increase in the conversion of the double bond is probably causing the gel effect to appear prematurely. This increases the steric effect between the molecules and makes it difficult to move the segments, which limits the diffusion and migration of molecules. Therefore, it eventually limits the efficiency of molecular polymerization [32].

In order to study the effect of type of photopolymerization (cationic or radical) and kinetic of photopolymerization on the cured films, the basic performance of coatings with different epoxy and acrylic group content was explored, as shown in Table 2. As expected, the tack-free time is the shortest for the cationic process of resins containing the highest content of epoxy groups and for the radical process of resins containing the highest content of acrylate double bonds, which is related to the photopolymerization rate of these systems. For the hardness test, with the decrease in the content of the epoxide groups in the case of the cationic process, the hardness gradually decreased. In turn, a decrease in the number of acrylate groups in the radical process resulted in obtaining the harder coatings. This is probably related to the conversion degree of the functional groups in the curing process. In the case of the cationic process, the reduction of the number of epoxy groups resulted in obtaining a lower conversion degree and thus a lower hardness in the coatings. The same effect was observed with the radical process because the reduced content of acrylate groups resulted in obtaining a lower degree of conversion and hardness. When comparing the presence of acrylate and methacrylate groups, the higher level of hardness of the coatings obtained in the cationic process is represented in the case of resins containing methacrylate groups, although they are not involved in the hardening process. This is possibly related to the higher rigidity of polymer chains containing methacrylate groups [39]. Another effect is obtained in the case of curing the coatings in the radical process, where the presence of methacrylate groups results in a lower hardness, which is probably related to achieving a lower degree of double bond conversion. The adhesion of the coatings cured in the cationic process was higher compared to the radical process and slightly higher for resins with an acrylate group compared to resins with a methacrylate group. The low rate of cationic polymerization relative to the radical polymerization, as well as the polymerization of acrylate versus methacrylate groups in the radical process, can lead to a lower cross-link density and stresses of the resulting networks, providing better coating adherence to the substrate and higher adhesion. All obtained coatings had a high gloss and were even higher in the case of the cationic process. The coatings obtained with the resin-containing acrylate groups had a higher yellowness compared to unmodified TMETGE or resin-containing methacrylate groups.

## 4. Conclusions

In this paper, a series of epoxy (met)acrylate prepolymers containing both epoxide and carbon-carbon double bond were successfully synthesized by means of an addition reaction of acrylic or methacrylic acid to the ether of trimethylolethane (TMETGE). The reaction course was monitored through titration methods (epoxy equivalent and acid value). The structures of prepolymers after the modifications were unambiguously confirmed using spectroscopic methods (FT-IR, ^1^H NMR, ^13^C NMR). It has been observed that the carboxyl-epoxide addition esterification proceeds in the presence of the catalyst, at a temperature of 90 °C. Moreover, one can easily control the degree of epoxy group conversion by varying the molar ratio of TMETGE to AA/MAA and the reaction time. Prepolymers developed herein are characterized by relatively low viscosity ranging from ~1100 mPa·s to ~4200 mPa·s, limited acid value (<20 mgKOH/g), and, most importantly, are capable of producing coatings by two different photopolymerization mechanisms: cationic and radical. The photoinitiated cationic or radical polymerization of the prepolymers was monitored by means of real-time infrared spectroscopy. It has been shown that cationic polymerization proceeds slower than radical polymerization and will lead to a lower degree of reaction of photoreactive functional groups. This may result from the difference in the rate of initiation rate of the photoinitiators used, the progressive stage of propagation, the viscosity of the system, the content of functional groups, and their reactivity. Moreover, the steric effect between the molecules caused by the presence of methyl groups in methacrylates made it difficult to move the segments, limiting the diffusion or migration of molecules. As a consequence, the efficiency of molecular polymerization is limited. This may also be related to the formation of different types of polymers (polyether or methacrylate backbone), differences in specific mobility of polymer chains, as well as reduced diffusion of active centers. As expected, the tack-free time is the shortest for the cationic process in resins containing the highest content of epoxy groups and for the radical process in resins containing the highest content of acrylate double bonds. The latter can be directly related to the photopolymerization rate of these systems. It has been shown that, despite the same backbone of the prepolymer molecules, cationic photopolymerization leads to coatings with better physicomechanical properties, i.e., hardness, adhesion, and gloss. This is probably related to the formation of a tightly cross-linked polyether structure.

## Figures and Tables

**Table 1:** Characteristics of TMETGE and TMETGE-AA/TMETGA-MAA prepolymers collected throughout the reaction.

Reaction Time (min)	AA						MAA					
	NV (%)	PAVs (mgKOH/g)	AAC (%)	EE (g/mol)	EGC (%)	η (mPa·s)	NV (%)	PAVs (mgKOH/g)	MAAC (%)	EE (g/mol)	EGC (%)	η (mPa·s)
	TMETGE											
	98.7			158.774		336	98.7			158.774		336
	TMETGE + 1AA						TMETGE + 1MAA					
0	87.27	106.1	0.0	165.334	0.1		84.18	111.7	0.0	190.055	0.2	
120	96.34	19.9	81.2	205.801	19.7		98.16	9.1	91.9	252.121	22.7	
240	98.56	2.2	97.9	233.678	29.3	1189	98.56	5.3	95.3	276.795	29.6	1344
	TMETGE + 2AA						TMETGE + 2MAA					
0	79.25	210.8	0.0	165.131	0.0		74.71	222.3	0.0	225.026	0.3	
120	93.06	59.6	71.8	258.223	36.0		94.22	42.2	81.0	389.916	42.5	
240							97.51	19.9	90.2	521.127	57.0	
300	98	15.6	96.3	404.444	59.2	4081						4291

NV—nonvolatile matter content, PAVs—partial acid number, AAC/MAAC—acrylic/methacrylic acid conversion, EE—epoxy equivalent, EGC—epoxy group conversion, η—viscosity.

**Table 2:** The basic properties of the cured coatings depending on the photopolymerization (C—cationic, R—radical).

Sample	H_max_^(1)^ (W/g)		R_p_^max (2)^ (s^−1^)		C ^(3)^ (%)		Tack-Free Time (s)		Hardness (s)		Adhesion		Gloss (GU)		Yellowness Index	
	C	R	C	R	C	R	C	R	C	R	C	R	C	R	C	R
TMETGE	36	7	3,37		34		11		74		0		154		4.3	
TMETGE + 1AA	24	24	2,08	73	13	57	12	15	50	41	0	1.5	169	140	11.5	10.1
TMETGE + 2AA	15	54	1,92	89	5	57	16	13	35	23	0,5	1	153	120	10.6	7.1
TMETGE + 1MAA	21	15	1,92	35	5	34	12	10	132	27	0	1.5	159	123	3.6	3.5
TMETGE + 2MAA	16	36	1,81	64	4	41	22	10	115	14	0,5	3	145	119	3.9	2.8

(1) Maximum heat flow peak; (2) maximum polymerization rate determined by RT-IR method; (3) conversion determined by RT-IR method;—no curing.

## Data Availability

The data presented in this study are available on request from the corresponding author.
